# Gut Microbiota and Development of Vibrio cholerae-Specific Long-Term Memory B Cells in Adults after Whole-Cell Killed Oral Cholera Vaccine

**DOI:** 10.1128/IAI.00217-21

**Published:** 2021-08-16

**Authors:** Denise Chac, Taufiqur R. Bhuiyan, Amit Saha, Mohammad M. Alam, Umme Salma, Nusrat Jahan, Fahima Chowdhury, Ashraful I. Khan, Edward T. Ryan, Regina LaRocque, Jason B. Harris, Firdausi Qadri, Ana A. Weil

**Affiliations:** a Department of Medicine, University of Washington, Seattle, Washington, USA; b Vaccine Science, International Center for Diarrheal Disease Research, Bangladesh, Dhaka, Bangladesh; c Department of Medicine, Harvard Medical School, Boston, Massachusetts, USA; d Division of Infectious Diseases, Massachusetts General Hospital, Boston, Massachusetts, USA; e Department of Immunology and Infectious Diseases, Harvard School of Public Health, Boston, Massachusetts, USA; f Department of Pediatrics, Harvard Medical School, Boston, Massachusetts, USA; g Division of Global Health, Massachusetts General Hospital for Children, Boston, Massachusetts, USA; University of California San Diego School of Medicine

**Keywords:** oral cholera vaccination, Shanchol, gut microbiota, memory B cell response

## Abstract

Cholera is a diarrheal disease caused by Vibrio cholerae that continues to be a major public health concern in populations without access to safe water. IgG- and IgA-secreting memory B cells (MBC) targeting the V. cholerae O-specific polysaccharide (OSP) correlate with protection from infection in persons exposed to V. cholerae and may be a major determinant of long-term protection against cholera. Shanchol, a widely used oral cholera vaccine (OCV), stimulates OSP MBC responses in only some people after vaccination, and the gut microbiota is a possible determinant of variable immune responses observed after OCV. Using 16S rRNA sequencing of feces from the time of vaccination, we compared the gut microbiota among adults with and without MBC responses to OCV. Gut microbial diversity measures were not associated with MBC isotype or OSP-specific responses, but individuals with a higher abundance of *Clostridiales* and lower abundance of *Enterobacterales* were more likely to develop an MBC response. We applied protein-normalized fecal supernatants of high and low MBC responders to THP-1-derived human macrophages to investigate the effect of microbial factors at the time of vaccination. Feces from individuals with higher MBC responses induced significantly different IL-1β and IL-6 levels than individuals with lower responses, indicating that the gut microbiota at the time of vaccination may “prime” the mucosal immune response to vaccine antigens. Our results suggest the gut microbiota could impact immune responses to OCVs, and further study of microbial metabolites as potential vaccine adjuvants is warranted.

## INTRODUCTION

Vibrio cholerae is the causal agent of cholera, an acute diarrheal disease that causes an estimated 91,000 deaths every year (1). Cholera is endemic in Sub-Sahara Africa and South East Asia, and nearly 3 million cases are reported annually (1). Over 200 serogroups of V. cholerae are found in the environment and two have caused epidemic disease in humans: V. cholerae O1 and, less commonly, O139. V. cholerae O1 are divided into serotypes Inaba and Ogawa that differ in the methylation of a terminal perosamine in the O-side chain of lipopolysaccharide (LPS), and both biotypes circulate in regions of cholera endemicity (2–5). After ingestion of V. cholerae-contaminated water or food, V. cholerae colonizes the small intestines and produces cholera toxin (CT) that is responsible for severe watery diarrhea and dehydration.

Oral cholera vaccines (OCVs) are an important tool in combating cholera and have been used in outbreaks in cholera-naive populations and in cholera-endemic areas (6, 7). Currently, the most widely used OCVs are killed whole-cell V. cholerae formulations without a recombinant CT subunit B (Shanchol, Shantha Biotechnics, India, and Euvichol or Euvichol-Plus, Eubiologics, South Korea). These vaccines consist of inactivated V. cholerae O139 and O1 strains of both Inaba and Ogawa serotypes, and require one or more doses to protect adults and children over 5 years of age (8–10). Vaccination with Shanchol usually generates a vibriocidal antibody response and circulating IgG and IgA antibodies to V. cholerae antigens (11–13). Plasma antibody levels wane quickly after vaccination and are not reliable indicators of response to vaccination. Memory B cells (MBC) are long-lived cells that circulate in the weeks after vaccination and can then be reactivated to produce antibodies rapidly after reexposure to an antigen (14). Studies of household contacts of patients with cholera indicate that measurable V. cholerae-specific MBC responses to the O-specific polysaccharide (OSP) component of the V. cholerae LPS correlate with protection against infection (15, 16). After vaccination with Shanchol, V. cholerae OSP-specific MBC responses are induced in some adults living in cholera areas of endemicity, such as Haiti and Bangladesh (17, 18). These responses peak 3 to 6 weeks after vaccination and wane over the period of 1 year (17, 18). Depending on the serotype and immunoglobin isotype, 0 to 67% of vaccine recipients develop detectable OSP MBC responses after vaccination (17, 18).

There are several hypotheses for this variation in immune response to OCV, including diet, preexisting immunity, and differences in the gut microbiome (19). Gut microbial communities have been correlated previously with immunological responses to oral vaccines; for example, Harris et al. reported that administration of antibiotics prior to live attenuated rotavirus vaccination correlated with immunologic response to vaccination (20). Another study of oral live attenuated typhoid vaccination found that differences in gut microbiota diversity at time of vaccination differentiated between persons with multiphasic versus late cell-mediated immune responses (21). Consistent with the concept of gut microbes at the site of vaccine absorption impacting vaccine response, small intestinal bacterial overgrowth has also been associated with a blunted immune response to live oral cholera vaccines (22, 23).

To investigate the relationship between the gut microbiota and responses to the OCV Shanchol, we analyzed the fecal microbiota at the time of vaccination and measured immune responses after vaccination in humans in Bangladesh. We identified gut microbial taxa that differentiate vaccine responders from nonresponders and characterized the baseline immune activation of responders compared to nonresponders by measuring cytokine responses to fecal metabolites in a human macrophage cell culture model.

## RESULTS

### Study enrollment and demographics.

Sixty-nine participants enrolled in a study designed to measure the immunogenicity of the Shanchol vaccine stored in ambient temperatures contributed feces for this study (24). Demographic and clinical characteristics of study participants are shown in Table 1. Study participants received one or two doses of Shanchol (at 14 or 30 days apart, see the Materials and Methods) and all of these regimens are known to be efficacious and immunogenic (8–10).

**TABLE 1:** Demographics and clinical characteristics of study participants

Characteristics	Participants, *n* = 69
Age (yrs)	
Mean	28.96
Range	18–44
Gender (%)	
Females	67 (97.1)
Males	2 (2.9)
Vibriocidal titer at day 0 (%)	
Ogawa ≥ 80	36 (52.2)
Ogawa < 80	33 (47.8)
Inaba ≥ 80	36 (52.2)
Inaba < 80	33 (47.8)

### Vibriocidal titer and V. cholerae OSP antibody responses.

**FIG 1:**
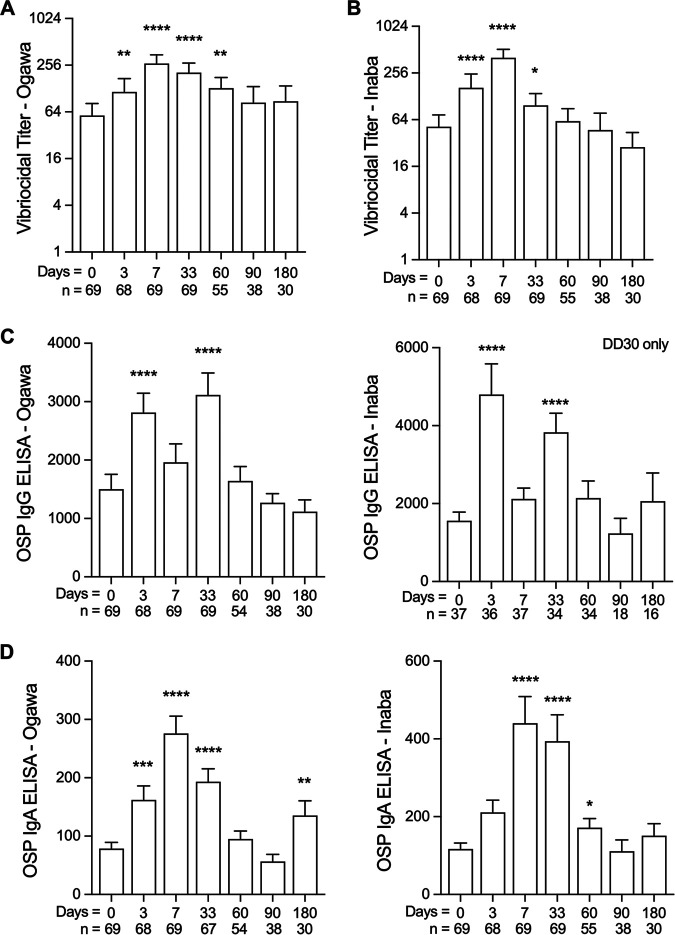
Vibriocidal titers and IgG and IgA antibody responses in study participants. (A and B) Ogawa (A) and Inaba (B) specific vibriocidal titers at day 0 through day 180 are shown. Day 0 is the date of administration for the initial vaccine dose. (C) IgG OSP-specific antibodies to Ogawa and Inaba serotypes. (D) IgA OSP-specific antibodies to Ogawa and Inaba serotypes. Vibriocidal titers are presented as geometric means with bars representing 95% confidence interval on a log_2_ scale. Mean values of OSP antibodies are shown with bars representing standard error of the mean (SEM). Mann-Whitney testing was performed and asterisks denoting statistically significant differences from baseline levels are shown; *, *P* < 0.05; **, *P* < 0.01; ***, *P* < 0.001; ****, *P* < 0.0001.

Similar to immune responses from prior cohorts, vibriocidal titers to V. cholerae O1 Ogawa and Inaba serotypes increased from baseline after vaccination (Fig. 1A and B) (18, 25). In total, 44 (64%) and 54 (78%) of the 69 vaccine recipients had a 4-fold or greater Ogawa- or Inaba-specific vibriocidal response, respectively, by day 7 (see Table S1 in the supplemental material). Individuals with elevated baseline vibriocidal titers had lower seroconversion rates by day 7 compared to individuals with lower baseline vibriocidal titers, as previously reported (Table S1) (18, 26). Another traditional measure of vaccine response to OCV is circulating IgG and IgA antibodies.

Plasma IgG antibodies targeting Ogawa and Inaba OSP were elevated above the baseline measurement by day 3 (*P* < 0.0001) and day 33 (*P* < 0.0001) and returned to baseline by day 60 (Fig. 1C). A similar pattern of response was observed for both serotypes of OSP-specific IgA responses. IgA antibodies to Ogawa OSP were increased by day 3 (*P* = 0.0002) and remained elevated to day 33 (*P* < 0.0001), while antibodies to Inaba OSP were significantly increased by day 7 (*P* < 0.0001) until day 60 (*P* = 0.03) (Fig. 1D). Similar results were also found when participants were stratified by high and low baseline vibriocidal titers. Independent of baseline vibriocidal titer status, IgG antibodies to OSP were significantly elevated by day 3 after the initial vaccination dose, while IgA antibodies to OSP were significantly elevated by day 7 (see Fig. S1 in the supplemental material).

### V. cholerae OSP-specific memory B cell responses.

**FIG 2:**
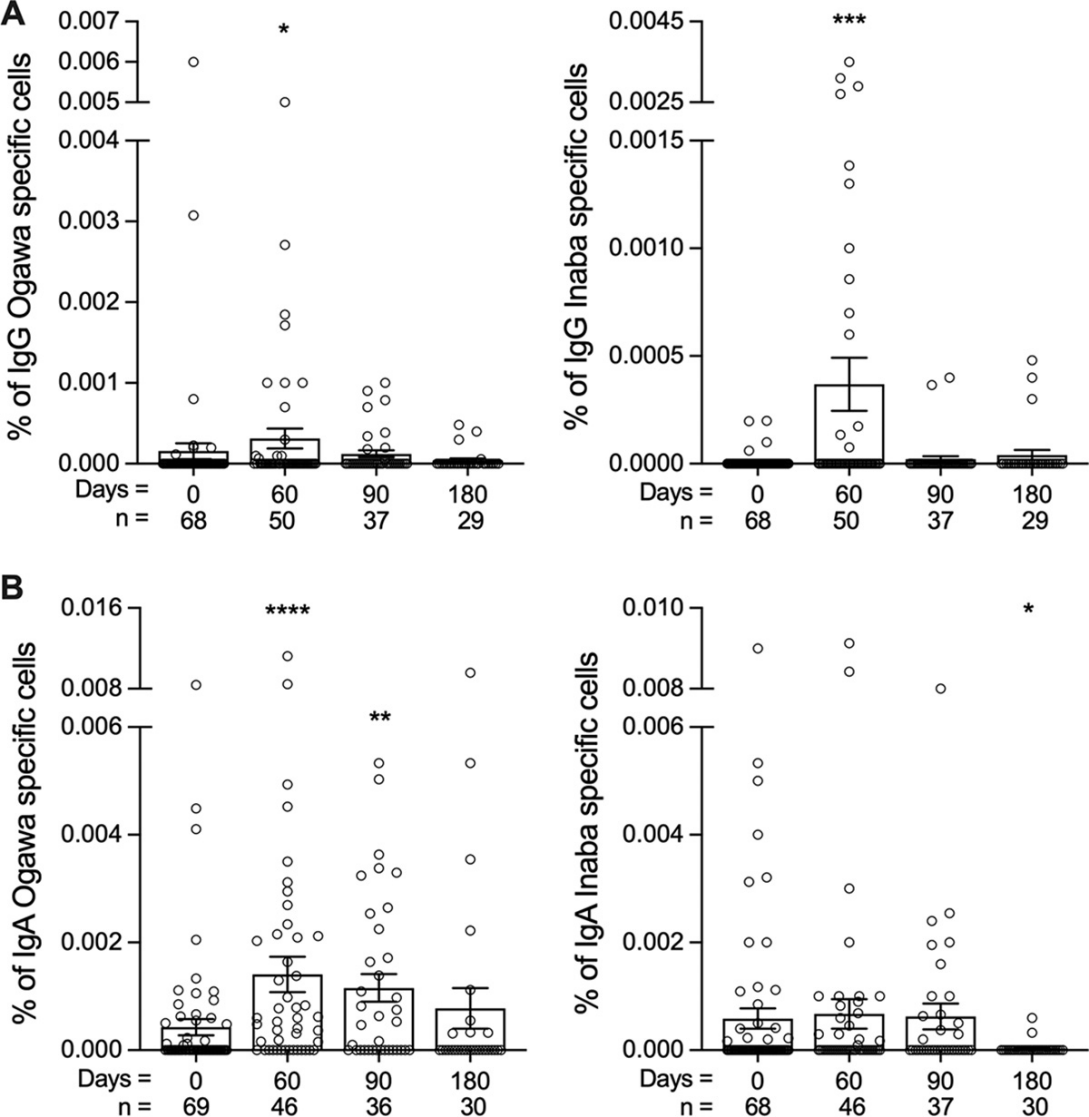
IgG and IgA OSP-specific memory B cell responses in study participants. (A and B) IgG (A) and IgA (B) Ogawa or Inaba specific MBC responses at day 0 through day 180. Day 0 is the date of administration for the initial vaccine dose. Data shown are means with bars representing SEM. Mann-Whitney testing was performed and asterisks denote statistically significant differences between follow up time points and baseline measurements; *, *P* < 0.05; **, *P* < 0.01; ***, *P* < 0.001; ****, *P* < 0.0001.

OSP-specific memory B cell (MBC) responses are correlated with longstanding immunity against V. cholerae (15, 16, 27). When a person with prior infection is reexposed to V. cholerae, long-lived OSP-specific MBCs are thought to rapidly differentiate, mature, and generate V. cholerae-specific OSP antibody responses that mediate protection from disease (28). After vaccination, IgG-specific MBC responses to Ogawa and Inaba OSP were significantly increased on day 60 and decreased at subsequent time points (Fig. 2A). IgA-specific MBC responses varied by serotype. There were 36% (25/69) and 26% (18/69) IgG MBC Ogawa and Inaba responders, respectively. While there was a significant increase in IgA-specific MBC to Ogawa OSP by day 60 (*P* < 0.0001), no change in IgA MBC response directed at Inaba OSP was observed (Fig. 2B). The frequency of IgA MBC responders was 62% (43/69) and 39% (27/69) for Ogawa and Inaba OSP, respectively.

### Gut microbiota composition before and after vaccination.

We studied the gut microbiota community composition and diversity before and after vaccination. In a subset of participants receiving two doses of Shanchol 14 days apart, microbial communities were analyzed at the time of initial vaccination (day 0) and follow-up days 7, 17, and 42. The number of sequences per sample used for analysis was normalized based on rarefaction curves (Fig. S2A). We measured the alpha and beta diversity of the gut microbiota between baseline and follow-up time points using inverse Simpson and Bray Curtis dissimilarity index. No significant changes in diversity or shifts in microbial community were found in the gut microbiota following vaccination in a subset of samples (Fig. S2B and C).

### Relationship between MBC responses and gut microbiota diversity.

**FIG 3:**
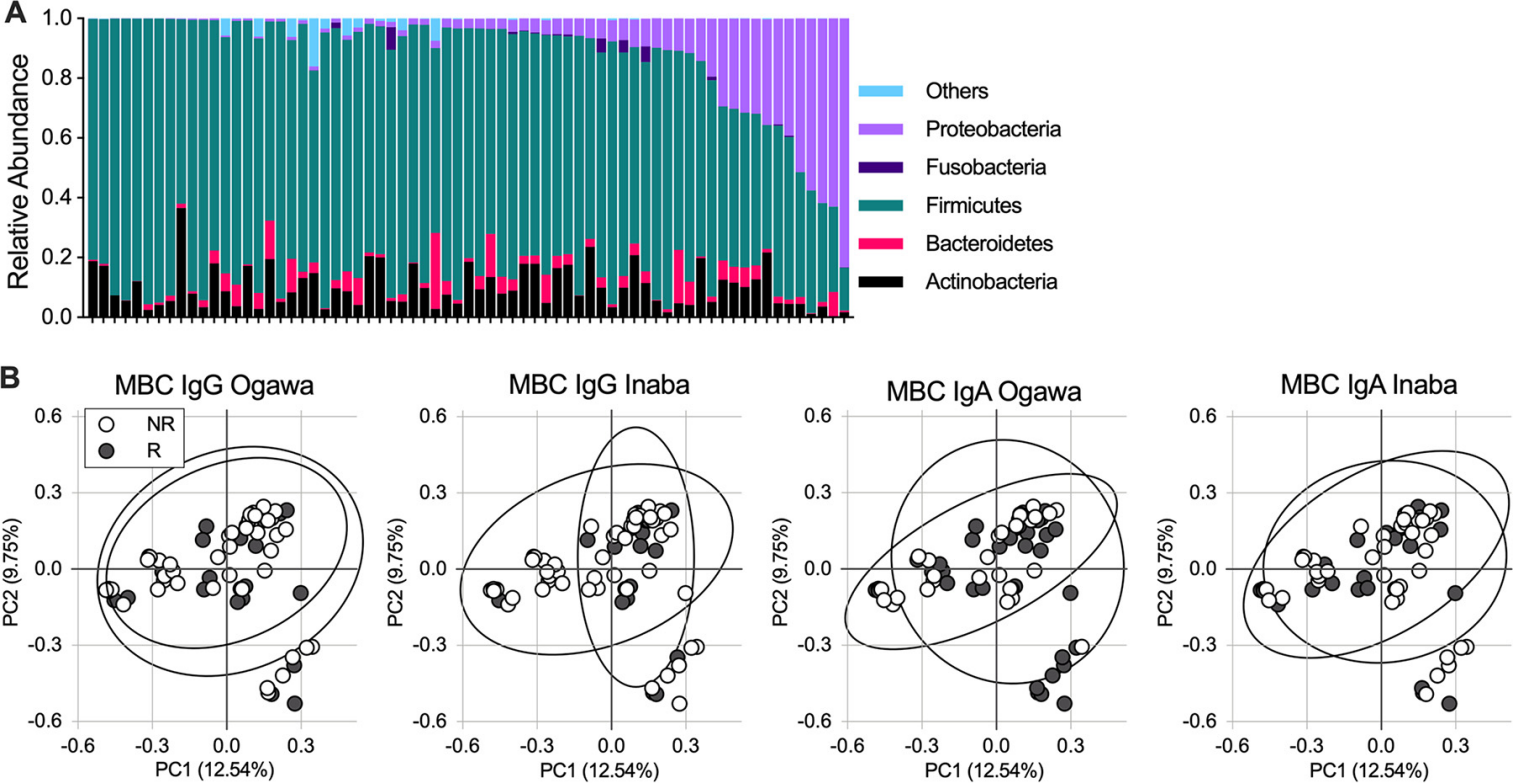
Microbiota composition at the time of vaccination in participants stratified by MBC response. (A) Phylum level abundance on the date of administration for the initial vaccine dose in each participant (baseline). (B) Principal-component analysis (PCoA) of beta diversity measured by Bray Curtis dissimilarity of MBC nonresponders (NR) and responders (R) by antibody isotype and V. cholerae serotype. Columns in (A) and dots in (B) represent a single participant with ellipses (B) representing the 95% confidence interval. Statistical testing of all four PCoA values using analysis of molecular variance (AMOVA) was not significant (*P* > 0.05).

Because our microbiota analysis combines data from persons who underwent three different vaccination regimens, we first compared the microbial diversity and community structure in the gut microbiota between dosing groups (Fig. S3). We found that the single dose vaccination group, with a small sample size of 12 participants, had low levels of *Proteobacteria* (Fig. S3A), with an increased inverse Simpson index compared to the other two groups (Fig. S3B). These findings were driven by an additional phylum present with small relative abundance in this group. Despite the variation in inverse Simpson index measures, all vaccine dosing groups had overlapping community structures by Bray Curtis measures of dissimilarity (Fig. S3C). Additionally, the gut microbiota alpha diversity was not correlated with age, baseline vibriocidal titer of <80 or ≥80, or presence of 4-fold change in vibriocidal titer after vaccination (Fig. S4). Because the vaccine dosing regimens used are all known to be effective in preventing cholera, and because the baseline diversity differences found between groups do not have a known relationship with how the gut microbiota would impact vaccination and may be influenced by small sample size when analyzed within vaccine arms, we combined the vaccination dosing groups to increase our likelihood of detecting a relationship between MBC responses and the gut microbiota, if this relationship is present. At baseline, the microbiota of the study participants was predominantly composed of phyla *Actinobacteria*, *Bacteroidetes*, *Firmicutes*, and *Proteobacteria* (Fig. 3A). Twelve of 69 participants had over 20% abundance of *Proteobacteria* and the overall mean abundance was 10%, consistent with prior studies of the gut microbiota in healthy Bangladeshi adults (29).

We next evaluated differences in phylum level abundance according to MBC response. There were no significant differences in phylum level abundance or alpha diversity as measured by inverse Simpson comparing nonresponder (NR) and responder (R) study participants (Fig. S5). Further analysis of beta diversity using principal-component analysis (PCoA) of Bray Curtis distance demonstrated overlap of NR and R bacterial communities, indicating similar community structures between NR and R participant microbiotas at time of vaccination (Fig. 3B). We found that the microbiota community structure at the time of vaccination and diversity measures did not differentiate NR from R participants, including by specific MBC immunoglobulin type.

### Bacterial communities differentially represented in MBC responders and nonresponders.

**FIG 4:**
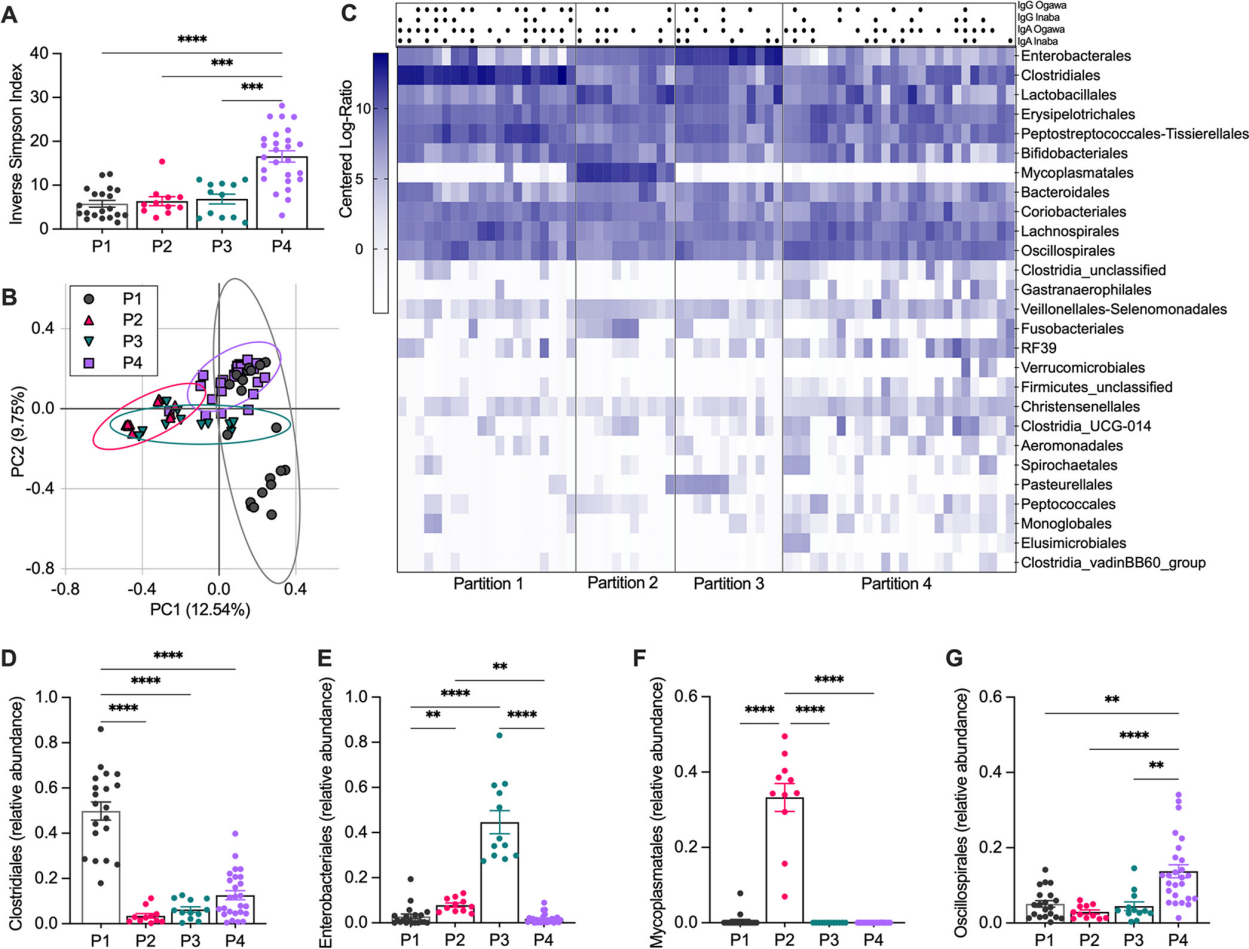
Microbiota communities present at the time of vaccination associated with differences in *Clostridiales* and *Enterobacterales*. (A and B) Partitions were created by unsupervised clustering of microbiota at baseline. Diversity of each partition was measured by inverse Simpson index (A) and PCoA using Bray Curtis dissimilarity (B). (C) Heat map of order-level bacteria as centered log-ratio abundance split by partitions. Black circles (•) denote MBC response and blank spaces denote lack of MBC response for the indicated measure. (D to G) Relative abundance of *Clostridiales* (D), *Enterobacterales* (E), *Mycoplasmatales* (F), and *Oscillospirales* (G) compared to total gut microbial taxa. Dots in (A and B) and (D to G) represent a single participant and data are shown as mean ± SEM. Ellipses in (B) represent 95% confidence intervals. Statistical testing in (A) and (D to G) are Kruskal-Wallis tests with multiple comparison adjustments; **, *P* < 0.01; ***, *P* < 0.001; ****, *P* < 0.0001.

To further investigate the microbiota at time of vaccination, we used an unsupervised modeling approach to identify bacterial taxa regardless of MBC characterization. Baseline taxa were fitted into unbiased community types using a Dirichlet multinomial mixtures model that clusters communities by the bacteria taxa present (30). Four microbial “partitions” resulted from modeling the baseline microbiota of vaccine participants at the bacterial order level (Fig. 4). Partition 4 (P4) had significantly higher diversity compared to the other three partitions (comparisons *P* < 0.0001, *P* = 0.007, and *P* = 0.0009, respectively, as shown in Fig. 4A), while analysis of the community structure with PCoA showed overlap of the partitions (Fig. 4B). A heat map of order-level taxa from each participant within the partitions demonstrates similar patterns between P2 and P3, with an increase in abundance of *Enterobacterales*, while P1 and P4 had increased *Clostridiales* (Fig. 4C). The bacterial taxa that contributed the most to the model for delineating the partitions were *Clostridiales*, *Enterobacterales*, *Mycoplasmatales*, and *Oscillospirales* (Fig. 4D to G; Fig. S6). *Clostridiales* was the top taxon in the model and was highly abundant in P1 (mean = 0.50 ± SD 0.18) compared to P2 (0.035 ± 0.04), P3 (0.062 ± 0.42), and P4 (0.13 ± 0.10) (*P* < 0.0001 for all comparisons, Fig. 4D). Conversely, P2 (0.079 ± 0.030) and P3 (0.44 ± 0.18) were characterized by high levels of *Enterobacterales* compared to P1 (0.028 ± 0.047; P1 versus P2: *P* = 0.009; P1 versus P3: *P* < 0.0001) and P4 (0.017 ± 0.021; P2 versus P4: *P* = 0.005; P3 versus P4: *P* < 0.0001) (Fig. 4E). P2 (0.33 ± 0.123) was defined by high levels of *Mycoplasmatales* that were nearly absent in the microbiota of persons classified in the other partitions (*P* < 0.0001 for all comparisons) (Fig. 4C and F). *Oscillospirales* was most abundant in P4 (0.138 ± 0.09) compared to P1 (*P* = 0.0011), P2 (*P* < 0.0001), and P3 (*P* = 0.002) (Fig. 4G). These data highlight the sources of variation and distinct microbial communities present in this population at the time before vaccination.

To investigate the relationship between MBC responses and the four partitions, each participant was labeled as an MBC responder or nonresponder for each immunoglobulin isotype and V. cholerae serotype (top row shown in Fig. 4C). All partitions had a 50% or greater proportion of responders to IgA Ogawa. Responses to IgA Inaba and responses to IgG of both serotypes were variable (full data shown in Table S2). Partition P1 had the highest percentage of responders overall, with more than 40% MBC response for each isotype and serotype (Fig. 4C; Table S2). As above, this partition was characterized by increased *Clostridiales*, and the most abundant taxa identified in this group were the *Sarcina* genus and the species *Clostridium sensu stricto* (as shown in Table S3). Although P4 had the highest alpha diversity values and community overlap with P1, this partition had a low frequency of MBC IgG responses (Ogawa 21%, and Inaba 27%) and the lowest IgA Inaba responses (35%) (Table S2). As we suspected from our beta diversity analyses, NR and R gut microbial communities did not separate neatly into partitions but were differentiated by specific bacterial taxa. We next investigated how fecal metabolites may impact vaccine response by querying the potential for NR and R feces to stimulate innate immune responses, independent of microbial diversity and partition assignment.

### Impact of responder compared to nonresponder fecal metabolites on innate immune responses of human-derived macrophages.

**FIG 5:**
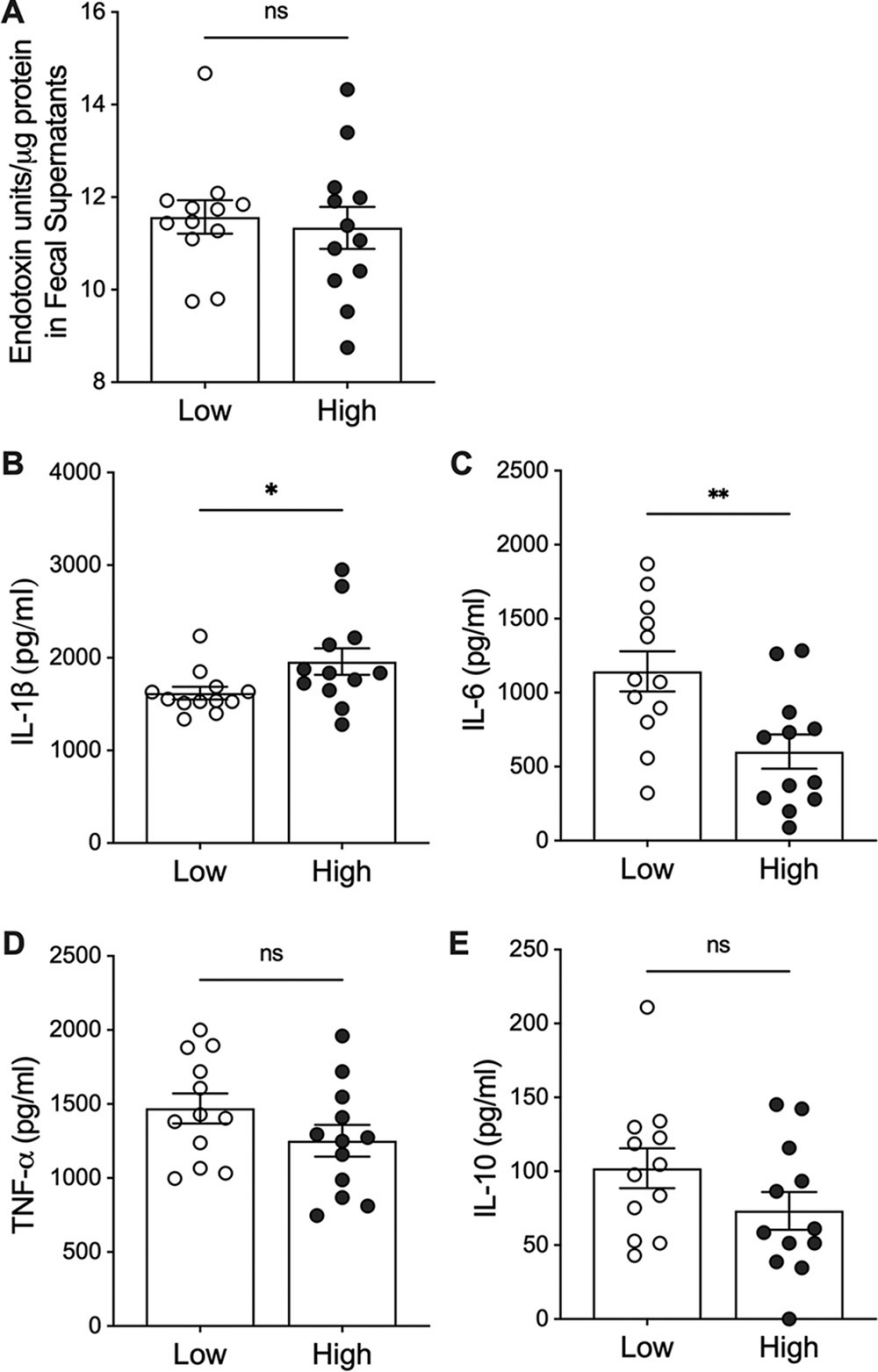
Cytokine responses of THP-1-derived macrophages following fecal supernatant stimulation. Fecal supernatants were extracted from a subset of individuals with high MBC responses (high) (*n* = 12) or low MBC responses (low) (*n* = 12) independent of vibriocidal titer or plasma antibody measures. (A) Measure of LPS in fecal supernatants by limulus amebocyte lysate assay expressed as endotoxin units. (B to E) Cytokine measurements in THP-1 supernatants after 24 h of incubation with fecal supernatants by ELISA for IL-1β (B), IL-6 (C), TNF-α (D), and IL-10 (E). Each dot denotes one participant’s fecal supernatant; data are shown as mean ± SEM and are representative of two independent experiments performed in duplicate. Mann-Whitney testing was used for statistical testing; *, *P* < 0.05; **, *P* < 0.01.

The development of long-term protective immune responses to V. cholerae infection is thought to be partially directed by the innate immune response to infection (31–34). We compared baseline fecal metabolites between study participants with high or low MBC responses to learn if baseline innate immune activation was associated with the postvaccination MBC response. We defined high responders as study participants with 3 or 4 detectable MBC responses (out of the four MBC measurements: IgA Ogawa, IgA Inaba, IgG Ogawa, and IgG Inaba), and low responders as participants with 1 or 0 detectable MBC responses. To assess the baseline fecal metabolite effects on innate immune responses independent of differential microbial taxa identified in our partition model, we selected high and low responders from all four of the above-described partitions. We hypothesized that the fecal metabolites of individuals that induced more inflammatory cytokines would have lower/absent MBC responses. Resuspension and filtering of feces removed large particles such as debris and bacteria, and supernatant was tested for sterility prior to application to cells and protein levels were normalized (see Materials and Methods). We first assayed endotoxin levels to assess for LPS, and these did not differ between high and low responders (Fig. 5A). Fecal supernatants were then applied and cultured with human THP-1-derived macrophages and innate cytokines were measured. Fecal extracts from participants with high MBC responses induced significantly higher levels of interleukin (IL)-1β compared to extracts from persons with low levels of MBC response (*P* = 0.037) (Fig. 5B), while low MBC participant extracts induced significantly higher levels of IL-6 (*P* = 0.0056) (Fig. 5C). There were no significant differences in the inflammatory cytokine tumor necrosis factor alpha (TNF-α) or the noninflammatory cytokine IL-10 (Fig. 5E and F). These differences indicate that fecal metabolites present at the time of vaccination polarize the innate immune responses.

**FIG 6:**
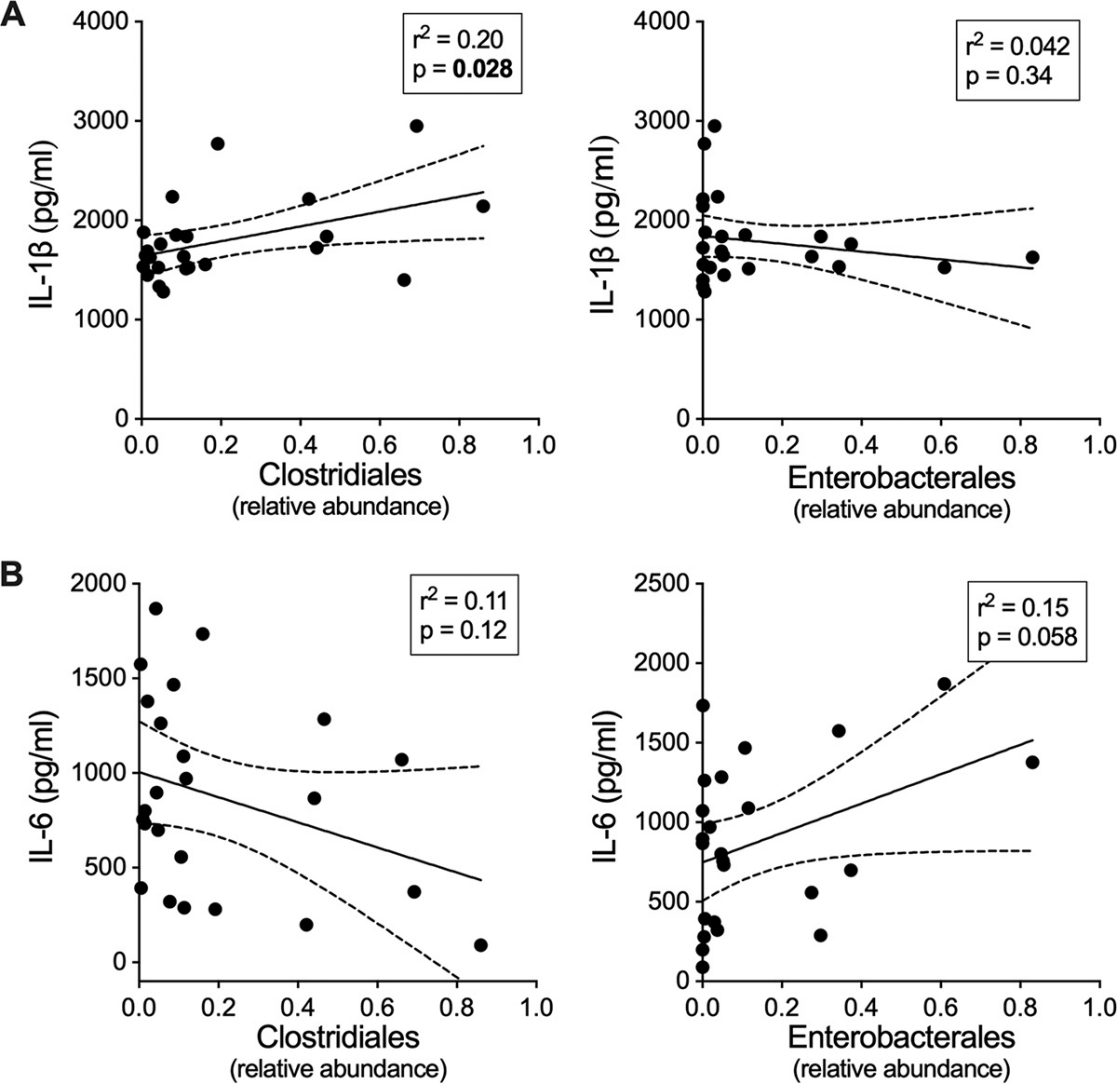
Relationship between fecal supernatant induced inflammatory cytokines and relative abundance of *Clostridiales* and *Enterobacterales*. (A and B) Correlation between IL-1β (A) and IL-6 (B) cytokine levels secreted from THP-1 cells and *Clostridiales* or *Enterobacterales* relative abundance in the fecal supernatants used for stimulation of THP-1 cells. Simple linear regressions with *r^2^* and *P* values are shown.

After identifying this difference in immune responses stimulated by fecal supernatants from high and low responders in our cell culture model, we correlated the bacterial taxa previously identified to stratify partitions with corresponding cytokine responses. Levels of IL-1β stimulated by fecal extracts were positively correlated with the relative abundance of *Clostridiales*, but not *Enterobacterales* (Fig. 6A). In contrast, while IL-6 levels were significantly different in cells stimulated by extracts from high compared to low MBC responders, IL-6 levels did not significantly correlate with either *Clostridiales* or *Enterobacterales* (Fig. 6B). Cytokine levels did not correspond with the amount of endotoxin in fecal supernatants (Fig. S7). In summary, specific bacterial groups, such as *Clostridiales*, were differentially abundant among study participants who developed higher MBC responses, and this taxon was also associated with generating differential cytokine responses in our fecal supernatant stimulation model, independent of endotoxin content. Together these results indicate that the presence of the metabolites of specific gut microbial taxa at the time of vaccination may influence immune responses to vaccination.

## DISCUSSION

Shanchol administered in a one- or two-dose regimen provides protection from V. cholerae infection in adults and the mechanisms of this protection remain under study (8, 35, 36). Antibodies that target the O-specific polysaccharide (OSP) and coat the sheath of the V. cholerae flagellum are known to correlate with protection from infection, most likely by inhibiting motility, thereby reducing V. cholerae virulence (16, 28, 37–39). Peripherally circulating memory B cells (MBC) that produce anti-OSP antibodies are generated after V. cholerae exposure or vaccination and correlate with protection from infection in human studies (15, 27). The MBC responses to Shanchol in cholera-naive and cholera-endemic populations vary, and a subset of persons receiving vaccine do not generate any OSP-specific MBCs (16, 17). Age, prior exposure to V. cholerae, enteric enteropathy, concurrent infection, and the gut microbiota have been proposed as factors that may impact OCV protective efficacy (9, 40, 41). Our study is the first to investigate the relationship between the gut microbiota and immune responses to OCV.

We found that the diversity of the gut microbiota at the time of OCV did not predict an increase in OSP-specific MBC responses. In prior studies, the relationship between microbial diversity and immune responses to oral vaccination has been mixed (21, 42–44). This suggests that the gut microbial impact on immune response to vaccination may be linked to the presence of specific strains, metabolites, or other factors that influence the intestinal environment, rather than a general measurement of gut microbial “health,” such as taxonomic diversity. Among our cohort of vaccine participants, the bacterial order *Clostridiales* differentiated the gut microbiota of persons most likely to have high compared to low MBC responses. *Clostridiales* are robust producers of short-chain fatty acids (SCFAs), which are fermentation products of anaerobic bacterial metabolism. Higher SCFA gut levels have been associated with positive effects on host metabolism and the health of the gut epithelium, and are known to modulate mucosal immunity (45–49). For example, SCFAs downregulate LPS-induced proinflammatory mediators, including IL-6, by inhibiting histone deacetylation activity in gut macrophages (50, 51). This is in agreement with our observation that less IL-6 was secreted in our human-derived macrophage model stimulated with fecal extracts from persons with more robust MBC responses.

In this study, the taxa most abundant in the *Clostridiales* group were the genus *Sarcina* and the *Clostridiales sensu stricto* species, which produce SCFAs. Both taxa have been previously associated with vegetarianism, and a plant-based diet is known to reduce gut oxidative stress and mucosal surface innate immune activation (52–57). Although we did not collect diet data on our study participants, the gut microbiota is known to be highly impacted by diet (58) and it is possible that some participants had low or no animal products in their diet, which may be due to availability and cost rather than lifestyle choice. Future studies of vaccine responses and the gut microbiota should consider collecting data on dietary habits as a host factor that could inform the gut microbiota-vaccine immune response relationship.

The gut microbiota associated with lower frequency of MBC responders was dominated by taxa from the group *Enterobacterales*. Strains from this taxon are typically aerobes and, in prior studies, have been associated with low-diversity microbiota and disruption of healthy gut flora (59–61). Abundant genera within *Enterobacterales* includes Klebsiella, Escherichia, and other species with pathogenic potential that are known to have more adhesins, siderophores, and antibiotic resistance genes than other taxonomic groups (62, 63). *Enterobacterales* also includes strains known to have inflammatory LPS and surface proteins that activate innate immune responses, contributing to baseline inflammation at the mucosal surface (64, 65). We hypothesize that a higher *Enterobacterales* abundance in the gut may result in a “blunting” of the innate immune response when vaccine antigens are presented. Innate immune responses generated by OCVs are relevant to protective immunity because they contribute to the development and growth of V. cholerae OSP-specific MBCs that later reactivate when a person is exposed to V. cholerae (31, 66–68). For example, IL-1β is a canonical innate inflammatory cytokine that promotes upregulation of innate effector proteins and stimulates development of T follicular helper cells that provide “help” to B cells in germinal centers (69, 70).

To explore the hypothesis that the gut microbiota of low vaccine responders was more likely to stimulate innate immune activation at baseline, we measured innate responses from human-derived macrophages stimulated with fecal extract containing microbial metabolites. We found that low MBC responder feces resulted in significantly more IL-6 secretion and less IL-1β secretion, independent of fecal endotoxin content. Both IL-1β and IL-6 stimulate production of acute phase proteins and facilitate the growth and development of neutrophils and B cell populations (71). Because IL-1β stimulates IL-6 expression, and these cytokines are often cosecreted in response to pathogen-associated molecular patterns, we were surprised that IL-1β and IL-6 secretion significantly differed in our model (72). IL-1β and IL-6 expression in macrophages both rely on nuclear factor kappa light-chain enhancer of activated B cells (NF-κB), a canonical innate immune pathway, but differ in their downstream activation and cytokine release pathways. IL-1β processing is dependent on cleavage by caspase-1, which is activated by assembly of the NOD-, LRR-, and pyrin domain-containing protein 3 (NLRP3) inflammasome, and release can be stimulated by specific bacterial toxins and extracellular ATP (73–76). Conversely, IL-6 processing and secretion is primarily regulated through posttranscriptional alterations by the AT-rich interactive domain containing protein 5a (77, 78), whose release occurs via LPS-mediated stimulation of macrophages. The opposing IL-1β and IL-6 secretion in our model in response to high or low responder fecal stimulation suggests that, in addition to microbial metabolites, diet- or host-derived small molecules in the intestinal environment may impact the downstream processing or release of IL-1β and IL-6 downstream of NF-κB stimulation. Fecal supernatant contains small proteins, SCFAs, bile metabolites, and other known immunomodulators such as flagellin that may impact these pathways. Future study of the small molecules in the fecal extract that could be responsible for polarizing responses to vaccine antigens are needed in order to identify active components for further testing as potential vaccine adjuvants.

OSP-specific MBC responses to both Inaba and Ogawa serotypes were studied because these two strains emerge and reemerge in cholera outbreaks in a cyclical pattern that is not well understood (79, 80). Immunologically, the degree of cross-protection between serotypes is incomplete and may be asymmetric (79, 81, 82). In this study, we did not find significant differences between Ogawa compared to Inaba OSP-specific MBC results. Our study was limited by small sample size and a primarily female study population. We did not collect blood group information on our study participants and there are conflicting reports about the impact of blood group O status on vaccine responses to whole-cell killed OCVs (83, 84). Our study population included individuals who underwent different dosing regimens of vaccination; however, all dose regimens are known to be protective in adults and elicit similar immune responses (8, 35, 36). The microbial analysis was performed using 16S rRNA sequencing and this provides only compositional data and does not give resolution on bacterial gene content, function, or expression between the gut microbiota of study participants. Overall, the microbiota composition in our population is consistent with other studies of healthy Bangladeshi adults (29). However, one vaccine group had a lower abundance of *Proteobacteria* than other groups. This may have been due to the small sample size of this vaccine group compared to the other groups. In our analysis of microbiota diversity over time after vaccination, we did not conduct daily sampling which would give increased resolution on the fluctuations of the microbiota over time. An additional limitation to this work is that the location of immunologic processing of OCV occurs in the small intestine (85); here, we use feces as a proxy for the gut microbiota of the gastrointestinal tract. Fecal extract used in *in vitro* experiments excluded large molecules and retains host- and diet- derived metabolites in addition to microbial-derived metabolites.

Our observations of the gut microbiota in adults receiving OCV suggest that gut microbes present at the time of vaccination may impact OSP-specific MBC responses and, specifically, that the taxon *Clostridiales* is correlated with more robust long-term MBC responses that are known to protect from reinfection. Identification of commensal microbes or bacterial metabolites that enhance vaccine responses could lead to candidate products for coadministration to boost vaccine efficacy.

## MATERIALS AND METHODS

### Study participants and sample collection.

Healthy adult volunteers between the ages of 18 and 45 were recruited for this study in Dhaka, Bangladesh (24). Participants consenting to the study were excluded if they reported fever, gastrointestinal symptoms, use of anti-diarrheal medication in past 7 days, major comorbidities, were immunocompromised, pregnant, or had previously received any OCV. Upon enrollment, participants were randomized into three arms as follows: the first group received a single dose of Shanchol, the second received two doses 14 days apart, and the third received two doses 30 days apart. Blood was collected on the day of vaccination (day 0) and subsequent time points. Single dose (SD) participants were followed on day 3, day 7, day 30, and day 90. Individuals receiving two doses 14 days apart (TD-14) participated in follow-up on day 3, day 7, day 17, day 42, day 90, and day 180 after the first dose. Participants receiving two doses 30 days apart (TD-30) had follow-up sampling on day 3, day 7, day 60, day 90, and day 180 after the first dose. Downstream analyses of immune responses combined all vaccine arms. Participants in each vaccine group were recruited simultaneously in the Mirpur area of Dhaka city between November and December 2012. Fecal samples were first collected the day of the initial vaccine dose, which we refer to in this study as “day 0” or “baseline.”

### Measures of vibriocidal antibodies.

To separate peripheral blood mononuclear cells (PBMCs) from plasma, blood samples were subjected to a density gradient centrifugation using Ficoll-Isopaque (Pharmacia, Piscataway, NJ). Measures of vibriocidal antibody responses in plasma were completed as previously described using V. cholerae O1 Ogawa (X-25049) and V. cholerae O1 Inaba (T-19479) (86). Vibriocidal titers were characterized as the reciprocal of the highest dilution with over 50% reduction in optical density compared to control wells without plasma. Seroconversion is defined as having a 4-fold or greater change in vibriocidal titer from the baseline measurement.

### Plasma OSP-specific IgG and IgA antibody responses.

Measures of OSP-specific IgG and IgA antibodies in plasma were conducted using enzyme-linked immunosorbent assay (ELISA) as previously described (86). Briefly, antigens V. cholerae O1 Ogawa OSP with bovine serum albumin (BSA, 1 μg/ml) or V. cholerae O1 Inaba OSP with BSA (1 μg/ml) were prepared in bicarbonate buffer (pH 9.6 to 9.8) and coated on 96-well polystyrene plates. V. cholerae O1 Ogawa and Inaba OSP and BSA conjugates were prepared as previously described using V. cholerae O1 El Tor PIC018 and PIC158 strains, respectively (87). Plasma was diluted 1:40 in 0.1% BSA with 0.05% Tween in phosphate-buffered saline (PBS) and 100 μl of diluted plasma was plated per well. Following incubation, horseradish peroxidase (HRP)-conjugated secondary antibodies for human IgG and IgA (Jackson ImmunoResearch, West Grove, PA) with a 1:1,000 dilution were applied, and plates were developed using ortho-phenylene diamine (Sigma, St. Louis, MO) in 0.1 M sodium citrate buffer (pH 4.5) and 0.01% hydrogen peroxide. Kinetic measurements were taken at an absorbance of 450 nm for 5 min with 14-s intervals. Values were recorded as milli-absorbance units/min. ELISA data were normalized using the ratio of the optical density of the test sample to the standard of pooled convalescent-phase sera (from previously infected patients with cholera) (88).

### Measures of memory B cell OSP-specific responses.

Memory B cells producing IgA and IgG specific for V. cholerae OSP were measured by using enzyme-linked immunospot assay (ELISPOT) as previously described (15, 89–91). Briefly, 5 × 10^5^ PBMCs/well were cultured in cell culture plates with complete medium (Rosewell Park Memorial Institute Medium [RPMI] 1640 containing 10% fetal bovine serum [FBS], 2 mM l-glutamine, 200 units/ml penicillin, 200 mg/ml streptomycin, and 50 mM beta-mercaptoethanol). For ELISPOT of MBCs, plates were coated using anti-human immunoglobulins, OSP, or keyhole limpet hemocyanin (KLH), blocked with RPMI 1640 containing 10% FBS, and then cells were applied for 5 h at 37°C in 5% CO_2_. Following incubation, plates were treated with HRP-conjugated goat anti-human IgA or IgG (Hybridoma Reagent Laboratory, Baltimore, MD) at a dilution of 1:500 and incubated overnight at 4°C. Plates were developed using 3-amino-9-ethyl carbazole and cells were quantified using a stereomicroscope. Measures of ELISPOT were calculated as the percentage of antigen-specific MBCs from the total MBCs of that specific isotype at the same time point. MBC values were considered valid if quality control criteria were fulfilled, as in prior studies. Briefly, data were included if (i) the ratio of total Ig MBC stimulated to total Ig MBC unstimulated was greater or equal to 3; (ii) unstimulated response for specific antigens was less than 3; and (iii) stimulated MBC response to KLH was less than 3 (15, 89, 92). Individuals receiving vaccination were classified as responders (R) if the MBC response at follow-up time points was higher than the baseline value measured at the time of vaccination, and nonresponders (NR) if there was no increase in MBC value at follow-up time points in comparison to the baseline value.

### Fecal microbiota DNA extraction and 16S sequencing.

Feces were collected in cryovials and frozen at −80°C. Fecal microbial DNA was extracted using Powersoil DNA isolation kit (Qiagen, Germany) with a modified protocol as previously described (93). Briefly, fecal sample was thawed on ice and approximately 100 mg of sample was added to the PowerBead tubes. The feces were treated with C1 solution, heated at 65°C for 10 min, 95°C for 3 min, and vortexed for 10 min at maximum speed. Samples were then treated and washed using C2 to C5 solutions provided by the Powersoil kit. The resulting DNA was eluted in DNase- and RNase-free water and quantified using NanoDrop ND-1000 (Thermo Scientific, Waltham, MA, USA). Sequencing for taxonomic characterization was performed targeting the V4 region of 16S rRNA amplified using 515F (5′-GTGCCAGCMGCCGCGGTAA-3′) and 806R primers (5′-GGACTACHVHHHTWTCTAAT-3′) (94) and paired-end reads were sequenced using the Illumina MiSeq platform (Illumina, San Diego, CA, USA).

### 16S rRNA data processing and analysis.

Raw files obtained as fastq files were curated using mothur v.1.44.1 following the MiSeq SOP (https://mothur.org/wiki/miseq_sop/, accessed 25 June 2020) (95). Briefly, paired-end reads were combined into contigs, sequences were screened for maxambig = 0 and maxlength = 275, and aligned to Silva 16S rRNA sequence data (silva.nr_v138.align). Aligned sequences were preclustered to allow up to a 2-bp difference and chimeras were detected and removed using VSEARCH (v2.13.3) (96). Sequences were classified using SILVA (v138) (97) with a confidence score greater than 80% and phylotyped to the family level using cluster.split(). Alpha diversity measures were calculated using the inverse Simpson index. Beta diversity measures was calculated using the Bray Curtis dissimilarity index and visualized using principal-coordinate analysis (PCoA) plots. Unsupervised probabilistic clustering of bacterial communities by Dirichlet multinomial mixtures was performed using the mothur command get.communitytype() using the default settings on the order level of operational taxonomic unites (OTUs) (30). The heatmap of order-level abundance was created with abundances normalized using center-log ratio transformation (98).

### Fecal supernatant.

Approximately 75 mg of feces was resuspended in 4 ml of sterile PBS (pH 7.0). Feces were homogenized by vortexing and incubating at room temperature for 30 min. After incubation, feces were centrifuged at 4,000 × *g* for 10 min at room temperature and supernatant was collected. The pH was normalized to 7.0 to 7.2 and filtered through a 0.2-μm polyethersulfone syringe filter. Fecal supernatants were plated on tryptic soy agar with 5% sheep’s blood (Thermo Scientific, Waltham, MA, USA) and incubated at 37°C aerobically for 48 h to ensure sterility. Protein levels in fecal supernatants were measured using a Bradford/Coomassie protein assay kit with BSA standard (Pierce/Thermo Scientific, Waltham, MA, USA). Fecal supernatants were stored at −20°C until use.

### Endotoxin assay.

Fecal supernatants were thawed on ice and diluted in sterile PBS. Endotoxin levels were measured using the Chromogenic Endotoxin Quant kit (Thermo Scientific, Waltham, MA, USA) according to the manufacturer’s instructions with pyrogen-free Eppendorf tubes, pipette tips, and sterile 96-well plates.

### THP-1-derived macrophage stimulation and cytokine ELISAs.

THP-1 monocytes were obtained from ATCC (TIB-202) and maintained in RPMI 1640 supplemented with l-glutamine, 25 mM HEPES, 10% FBS, and Pen-Strep with 5% CO_2_ at 37°C. To induce differentiation to a macrophage phenotype, cells were seeded in 48-well tissue culture treated plates with 50 ng/ml phorbol 12-myristate 13-acetate (Invivogen, San Diego, CA, USA) for 72 h. Prior to treatment with fecal supernatants, the adherent THP-1-derived macrophages were washed 3 times with sterile PBS. Fecal supernatants were normalized for protein as described above and supernatants were applied to cells at a quantity of 10 μg/ml of protein for a total volume of 400 μl per well. After 24 h, cell supernatant was collected and assessed for cytokine production using ELISA. Cytokines were measured using human IL-1β, IL-6, tumor necrosis factor alpha (TNF-α), and IL-10 DuoSet kits (R&D Systems, Minneapolis, MN, USA) according to the manufacturer’s instructions.

### Ethics statement.

All studies involving OCV participants were approved by the Research Review and Ethics Review Committee of the International Centre for Diarrheal Disease Research, Bangladesh (icddr,b) in Dhaka, Bangladesh, and the Institutional Review Board of the Massachusetts General Hospital and the University of Washington. The vaccine Shanchol is prequalified by the WHO for use in populations at risk for cholera, such as persons living in Dhaka, Bangladesh (99). Written informed consent was obtained from all participants in this study.

### Statistical analysis.

All figures and statistical testing were generated using Prism (GraphPad, San Diego, CA version 9.0.2) or R (version 4.0.2). PCoA ellipses represent the 95% confidence interval. Statistical testing for differences in immunological responses to vaccine compared to day 0 was performed using Mann-Whitney testing with two-tailed *P* values. Mann-Whitney testing was also performed on alpha diversity measures of 16S microbiota data between NR and R groups. Comparison of alpha diversity and bacterial abundances of partitions was performed with Kruskal-Wallis test with Dunn’s multiple comparisons. Cytokine measurements of treated THP-1-derived macrophages were tested using Mann-Whitney testing and simple linear regression analysis was performed comparing bacterial abundances to cytokine levels. *P* values lower than 0.05 were considered statistically significant.

### Data availability.

The microbiome sequencing data has been deposited at BioProject under accession number PRJNA742046.
