# Supplementary material for: Gut Microbiota and Development of Vibrio cholerae-Specific Long-Term Memory B Cells in Adults after Whole-Cell Killed Oral Cholera Vaccine
Source: Infect Immun. 2021 Aug 16;89(9):e00217-21. doi: 10.1128/IAI.00217-21 (PMC8370679; doi:10.1128/IAI.00217-21)
Supplement: Supplemental file 1 — Supplemental material. Download IAI.00217-21-s0001.pdf, PDF file, 1.7 MB [file iai.00217-21-s0001.pdf]

**TABLE S1** Vibriocidal titer seroconversion of study participants by day 7 after the initial dose. Seroconversion is defined as a four-fold or greater increase in vibriocidal titer on day 7 compared to the day 0 measurement.

| **Seroconverted by Day 7** | **Ogawa** [percentage (number)] | **Inaba** [percentage (number)] |
|---|---|---|
| All vaccinees | 64 (44/69) | 78 (54/69) |
| Baseline Ogawa vibriocidal titers < 80 | 91 (30/33) | 91 (30/33) |
| Baseline Ogawa vibriocidal titers ≥ 80 | 39 (14/36) | 67 (24/36) |
| Baseline Inaba vibriocidal titers < 80 | 79 (26/33) | 97 (32/33) |
| Baseline Inaba vibriocidal titers ≥ 80 | 50 (18/36) | 61 (22/26) |

**TABLE S2** Memory B cell responder frequency within partitions.

| Partition | Total | IgG Ogawa n (%) | IgG Inaba n (%) | IgA Ogawa n (%) | IgA Inaba n (%) |
|---|---|---|---|---|---|
| **P1** | 20 | 12 (60) | 8 (40) | 15 (75) | 9 (45) |
| **P2** | 11 | 3 (27.3) | 1 (9.1) | 6 (54.5) | 4 (36.4) |
| **P3** | 12 | 4 (33.3) | 2 (16.7) | 6 (50) | 5 (41.7) |
| **P4** | 26 | 6 (21.1) | 7 (26.9) | 16 (61.5) | 9 (34.6) |

**TABLE S3** (see attached dataset) Most abundant OTUs within this cohort by total number of reads (top 100). Highlighting indicates one of the top four orders delimiting the four partitions: green for Enterobacterales, blue for Clostridiales, orange for Mycoplasmatales, and yellow for Oscillospirales. Values in parentheses (##) represent percentage of sequences within OTU with indicated classification; unless specified, all classifications are 100% or (100).

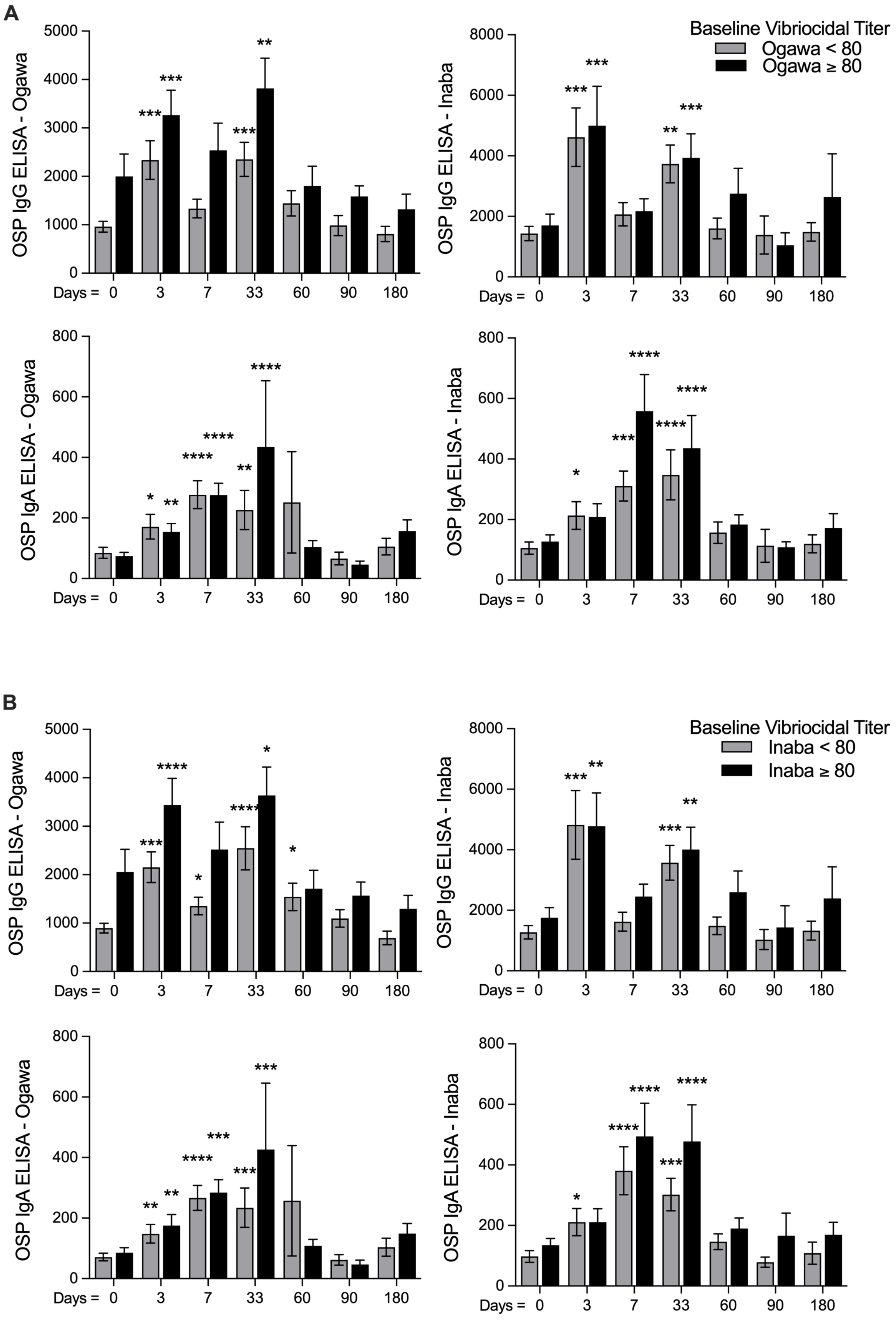

A
OSP IgG ELISA - Ogawa
OSP IgG ELISA - Inaba
OSP IgA ELISA - Ogawa
OSP IgA ELISA - Inaba
Baseline Vibriocidal Titer
Ogawa < 80
Ogawa ≥ 80
Days = 0 3 7 33 60 90 180
B
OSP IgG ELISA - Ogawa
OSP IgG ELISA - Inaba
OSP IgA ELISA - Ogawa
OSP IgA ELISA - Inaba
Baseline Vibriocidal Titer
Inaba < 80
Inaba ≥ 80
Days = 0 3 7 33 60 90 180

**FIG S1** IgG and IgA antibody responses in study participants stratified by baseline vibriocidal titer for (**A**) Ogawa and (**B**) Inaba. Mean values of OSP antibodies are shown with bars representing standard error mean. Mann-Whitney testing was performed and asterisks denote statistically significant differences from baseline levels. *p<0.05; **p<0.01; ***p<0.001; ****p<0.0001.

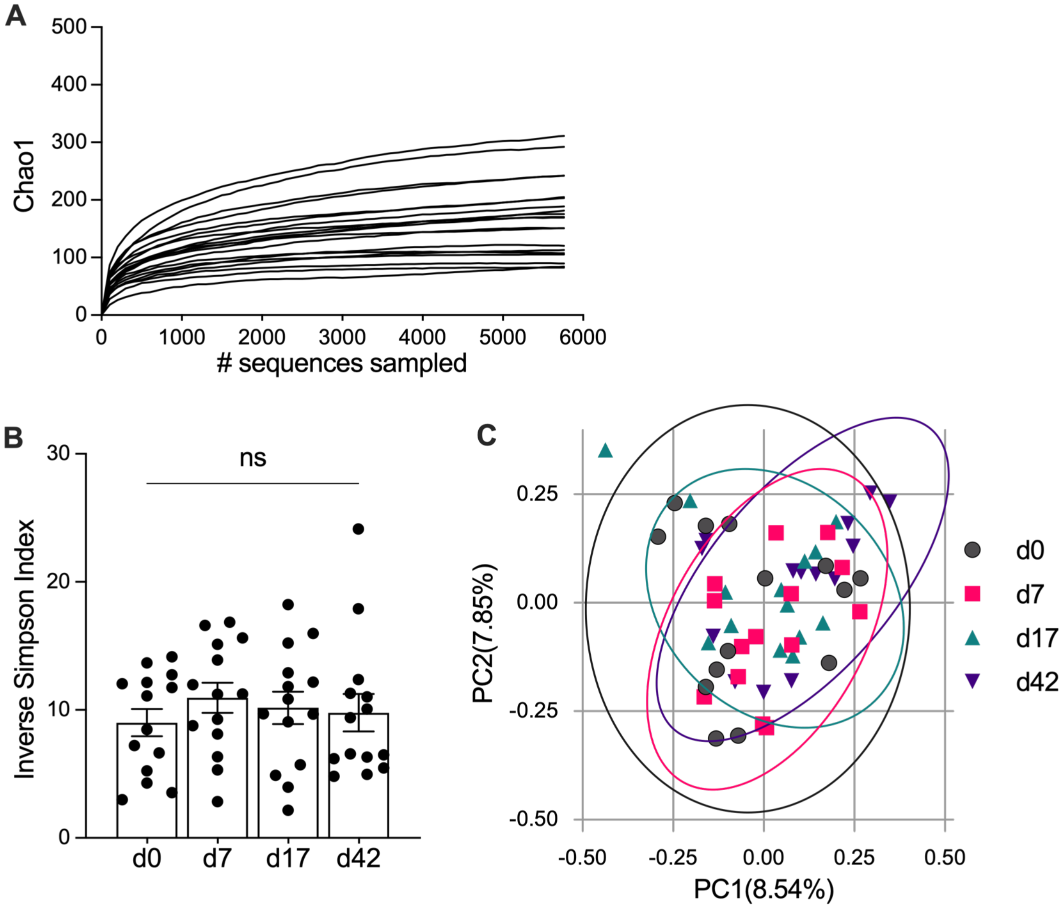


**FIG S2** Microbiome diversity over time. Day 0 (d0) indicates baseline microbiota at the time of vaccination and day 7, 17, and 42 represent dates of fecal collection after the initial dose. (**A**) Rarefaction curve of 16S rRNA sequencing results using the Chao1 index for diversity based on the abundance of OTUs in a community per 1000 sequencing reads. Plateauing of this curve beyond 5000 reads indicate that an increased number of sequences obtained per sample does not reveal additional microbial taxa in this cohort. (**B**) Inverse Simpson Index of microbiota in a subset of participants who received two doses 14 days apart (n=14) and (**C**) PCoA of Bray Curtis Dissimilarity of vaccine participants over time (d=days following vaccination with initial dose) in this subset. Statistical testing for (B) is one-way ANOVA with Tukey’s multiple

comparisons with mean ± standard error mean; $p>0.05$. Each symbol represents one individual and ellipses for (C) are drawn on a 95% confidence interval.

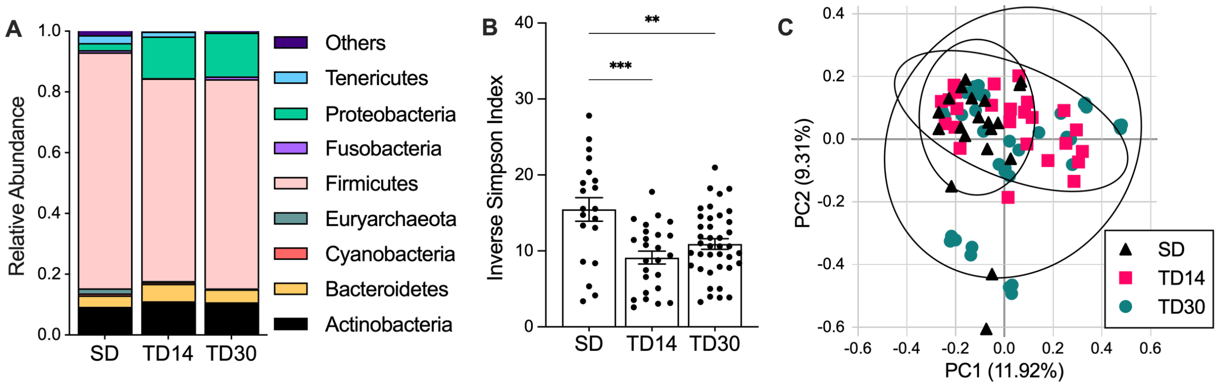


**FIG S3** Microbiome composition and diversity by vaccine arm. **(A**) Phylum-level abundance of composition, (**B**) Inverse Simpson Index, and (**C**) PCoA of Bray Curtis Dissimilarity of microbiome at time of vaccination with initial dose by vaccine arm. SD = single dose. TD = two doses, 14 or 30 days apart. Sample size is n=12 for SD, n=20 for TD14, and n=37 for TD30. (A) Statistical testing by Kruskal-Wallis test per phylum-level abundance with multiple comparisons, p>0.05. (B) Statistical testing by one-way ANOVA with Tukey's multiple comparisons, **p<0.01; ***p<0.001 with data shown as mean ± standard error mean. Each symbol represents one individual and ellipses in (C) are drawn on a 95% confidence interval.

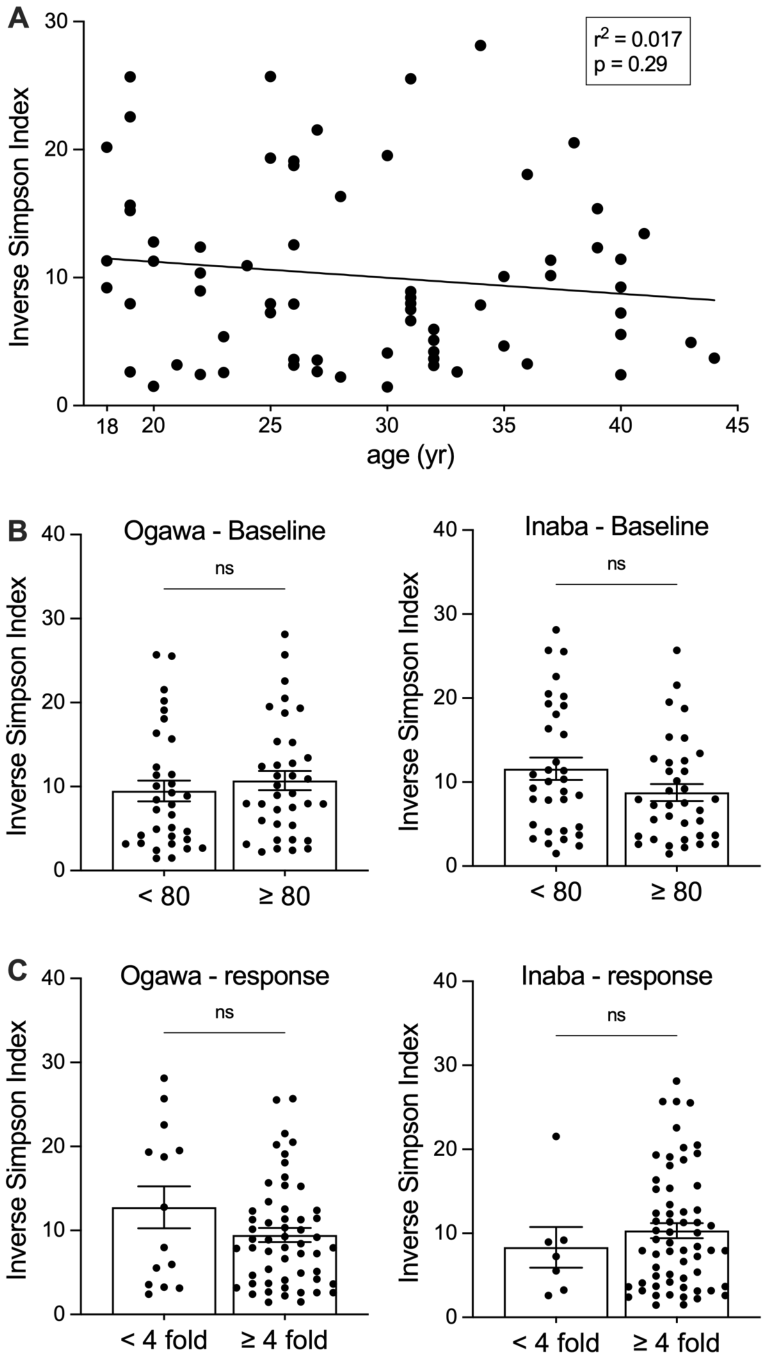


**FIG S4** Microbiome diversity by age and vibriocidal titers. Inverse Simpson Index measure of diversity by (**A**) age in years, (**B**) baseline vibriocidal titers less than 80 or greater than or equal to 80, and (**C**) vibriocidal titer fold change in response to vaccination. Statistical testing for (A) is simple linear regression and (B-C) is an

unpaired t-test, $p>0.05$ for all comparisons, with data shown as mean ± standard error mean.

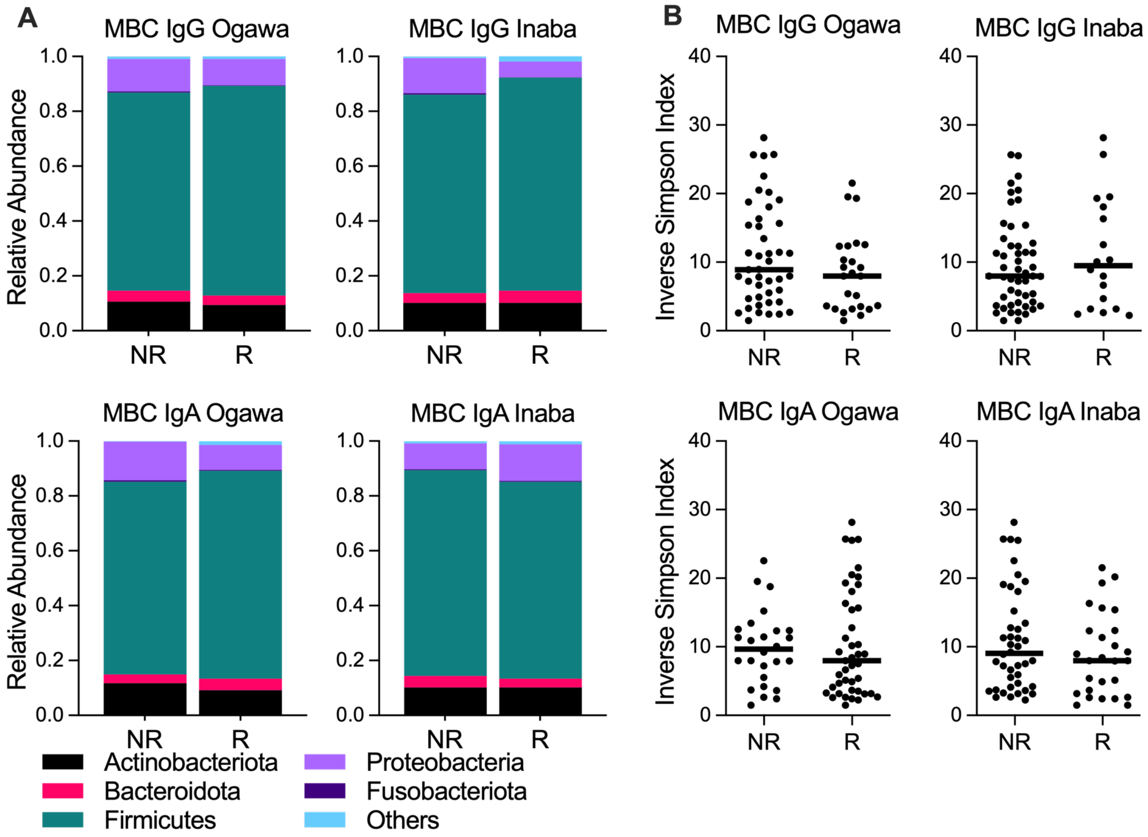


**FIG S5** Microbiome composition by MBC isotype and antigen type. (**A**) Phylum-level composition and (**B**) alpha diversity measured by Inverse Simpson Index separated by IgG- and IgA-specific MBC response to Ogawa and Inaba OSP antigens. NR = nonresponder and R = responder.

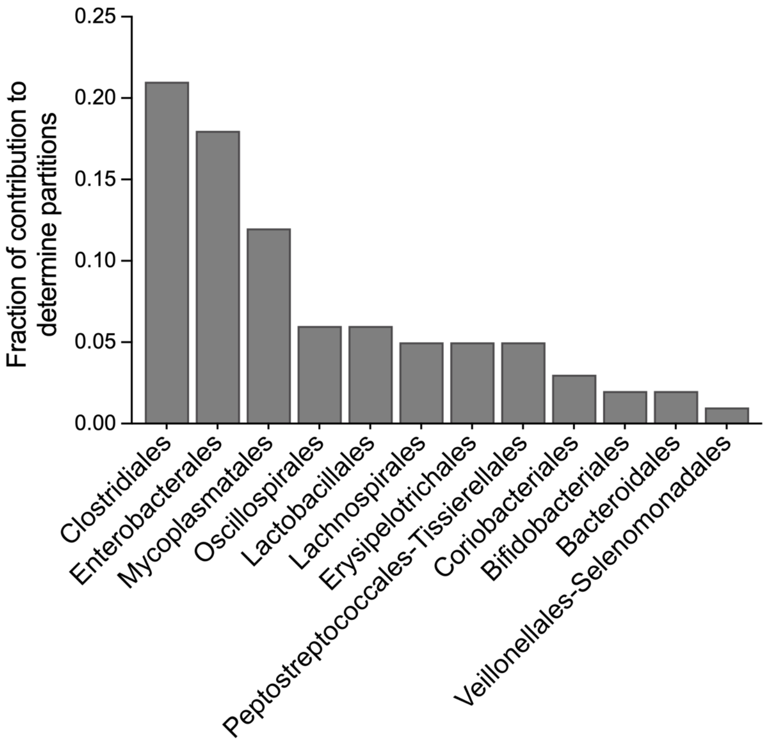


**FIG S6** Top bacterial groups contributing to modeling of microbiota data into partitions using Dirichlet multinomial mixtures.

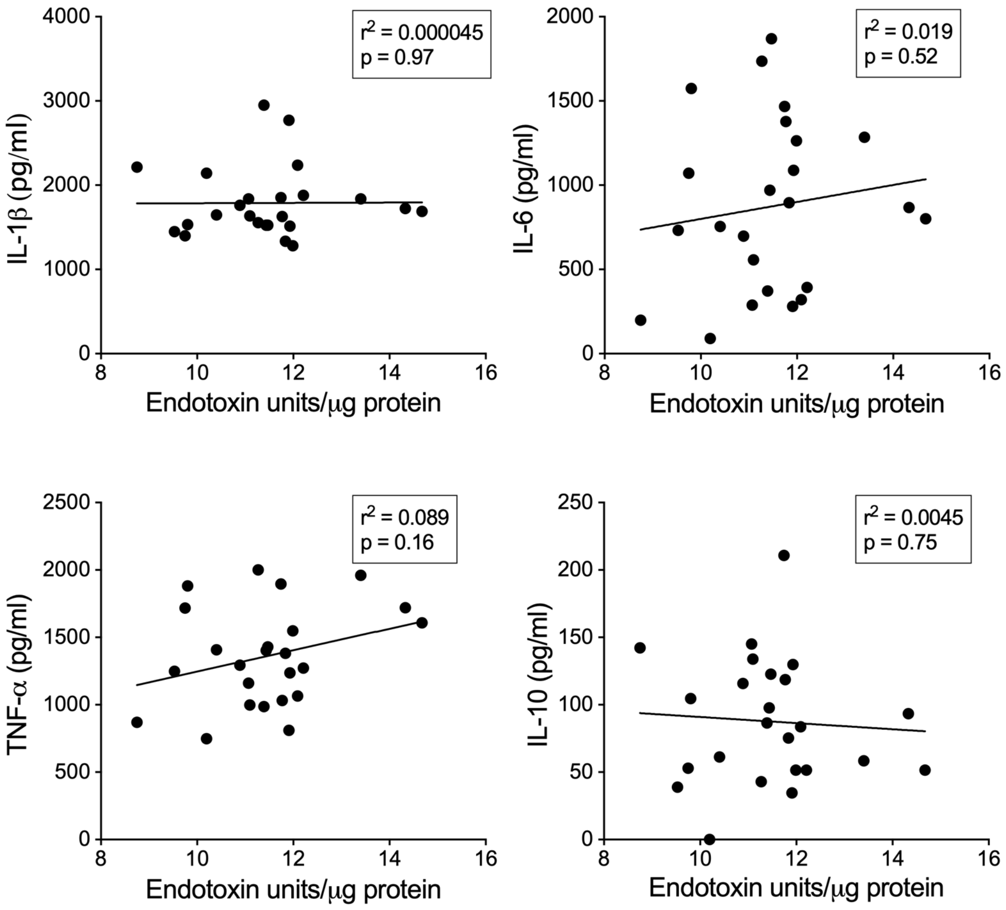


**FIG S7** Correlation between endotoxin units in fecal supernatants and induction of cytokines in THP-1 derived macrophages. Statistical testing shown is simple linear regression.
